# The Current State of Research, Challenges, and Future Research Directions of Blockchain Technology in Patient Care: Systematic Review

**DOI:** 10.2196/18619

**Published:** 2020-07-20

**Authors:** Polina Durneva, Karlene Cousins, Min Chen

**Affiliations:** 1 Department of Information Systems & Business Analytics Florida International University Miami, FL United States

**Keywords:** blockchain, health information technology, systematic review, security, privacy, interoperability, health outcomes

## Abstract

**Background:**

Blockchain offers a promising new distributed technology to address the challenges of data standardization, system interoperability, security, privacy, and accessibility of medical records.

**Objective:**

The purpose of this review is to assess the research on the use of blockchain technology for patient care and the associated challenges and to provide a research agenda for future research.

**Methods:**

This review followed the Preferred Reporting Items for Systematic Reviews and Meta-Analysis guidelines. We queried the Cumulative Index of Nursing and Allied Health Literature (CINAHL), PubMed, Excerpta Medica dataBASE (EMBASE), and Web of Science databases for peer-reviewed research articles published up to December 2019 that examined the implementation of blockchain technology in health care settings. We identified 800 articles from which we selected 70 empirical research articles for a detailed review.

**Results:**

Blockchain-based patient care applications include medical information systems, personal health records, mobile health and telemedicine, data preservation systems and social networks, health information exchanges and remote monitoring systems, and medical research systems. These blockchain-based health care applications may improve patient engagement and empowerment, improve health care provider access to information, and enhance the use of health care information for medical research.

**Conclusions:**

Blockchain health information technology (HIT) provides benefits such as ensuring data privacy and security of health data, facilitating interoperability of heterogeneous HIT systems, and improving the quality of health care outcomes. However, barriers to using blockchain technology to build HIT include security and privacy vulnerabilities, user resistance, high computing power requirements and implementation costs, inefficient consensus algorithms, and challenges of integrating blockchain with existing HIT. With 51% of the research focused on medical information systems such as electronic health record and electronic medical record, and 53% of the research focused on data security and privacy issues, this review shows that HIT research is primarily focused on the use of blockchain technologies to address the current challenges HIT faces. Although Blockchain presents significant potential for disrupting health care, most ideas are in their infancy.

## Introduction

### Background

There is a growing need to integrate health care information across a range of uses and stakeholders and to secure such data from unauthorized breaches, while making it easier for patients to access patient data. Blockchain is a distributed technology that has the potential to address data standardization challenges, system interoperability, and accessibility of medical records to support a more secure, patient-centric approach to health care information systems.

Blockchain is a secure and immutable transaction ledger [1,2], which is distributed in a decentralized manner across all computing devices that are part of the blockchain infrastructure [3]. Blockchain’s decentralized design facilitates peer-to-peer–based network transactions between users without the need for a trusted third party. Although more commonly used in decentralized financial applications such as cryptocurrencies and initial coin offerings (ICOs), blockchain’s advanced features (eg, consensus mechanisms, digital signatures, and hash chains) promise to address the unique challenges health information systems commonly face, such as poor security, privacy, efficiency, and interoperability.

Blockchains can be classified into 2 types: permissionless and permissioned blockchains [4]. The permissionless blockchain is open to the public, allows anyone to join the blockchain without approval, and usually provides an economic incentive for participating in the blockchain. Most known cryptocurrency blockchains are public, such as Bitcoin and Litecoin. A permissioned blockchain incorporates access control mechanisms to restrict user access. Permissioned blockchains are further classified as private or consortium blockchains based on their governance structure. A private blockchain is managed by a single organization and is usually used in enterprise solutions. The consortium blockchain is semiprivate, has a controlled user group, and works across different organizations. Compared with the public blockchain, permissioned blockchains are restrictive, and a central authority grants access to the blockchain. Therefore, permissioned blockchains lose some of the advantages of decentralization, but are more effective in securely sharing and managing real-time data among participating health care stakeholders. Embleema and the Synaptic Health Alliance are examples of health care blockchain consortia that allow selected health care stakeholders, including patients, advocacy groups, life sciences companies, payers, authorities, and care centers, to form nodes on the networks and manage health data securely, without a central authority.

Researchers are recognizing blockchain’s potential as a disruptive technology in health care and have begun to conduct research on how to leverage blockchain. We seek to understand what progress has been made in blockchain health care research and which problems researchers are addressing and encountering in use case implementations. We answer these questions by conducting a literature review and synthesizing the empirical blockchain health care research. The format of this systematic review adhered to the literature review standards of the review methodology, Preferred Reporting Items for Systematic Reviews and Meta-Analyses (Multimedia Appendix 1) [5].

This analysis shows that health care researchers are focusing on interoperability and platform issues and are exploring opportunities to use blockchain to enhance privacy and security and improve data integrity and transparency. We also identify the different types of blockchain health information technology (HIT) and discuss their implications for patient engagement and empowerment, provider access to personal health information (PHI), and medical or clinical research. We discuss the barriers and challenges of using and implementing blockchain HIT and propose new research directions.

The rest of the paper is organized as follows. First, in the *Methods* section, we describe the search process and selection criteria. In the *Results* section, we summarize our findings by highlighting the HIT challenges that blockchain addresses, the different types of blockchain-based HIT, how blockchain HIT research has evolved over time, and barriers and challenges to implementation. In the *Discussion* section, we suggest potential areas of research. In the *Conclusion* section, we end our review by summarizing and highlighting the implications of our key findings.

### Objective

The purpose of this review is to evaluate the current literature on the application of blockchain technology in health care with a focus on patient care, to assess the current state of research, the associated challenges, and potential areas for future research. In accordance with our objectives, the research questions are as follows:

1. What current HIT challenges does blockchain address?
2. What are the predominant applications for patient care in blockchain HIT research?
3. How has blockchain HIT research evolved over time?
4. What health care activities are impacted by current blockchain HIT research?
5. What are the challenges associated with blockchain-based HIT implementations?

## Methods

### Study Identification and Selection

Eligible papers were published in academic peer-reviewed journals and conference proceedings in English.

We searched bibliographic databases such as Web of Science, PubMed, Cumulative Index of Nursing and Allied Health Literature (CINAHL), and Excerpta Medica dataBASE (EMBASE). As PubMed, CINAHL, and EMBASE focus mostly on medical content, we used the search term “blockchain” to query these databases to execute a broad search so as not to miss important results. As the Web of Science provides content related to a broader range of topics, we used the search terms “blockchain” AND “health” OR “blockchain” and “medical” to query this database. We searched for all papers published up to December 12, 2019. The searches identified 800 potential articles: Web of Science, 298; PubMed, 184; CINAHL, 129; and EMBASE, 189. To validate our results, we conducted an alternative search using the search terms “blockchain” AND (health OR medic OR biomedic OR clinic). The alternative search terms came from prior research [6]. This alternative search identified only 652 potential articles and no new results, compared with the 800 potential articles initially identified: Web of Science, 264; PubMed, 146; CINAHL, 9; and EMBASE, 151.

### Data Extraction and Analysis of Types of Blockchain Health Care Applications and Benefits

**Figure 1:**
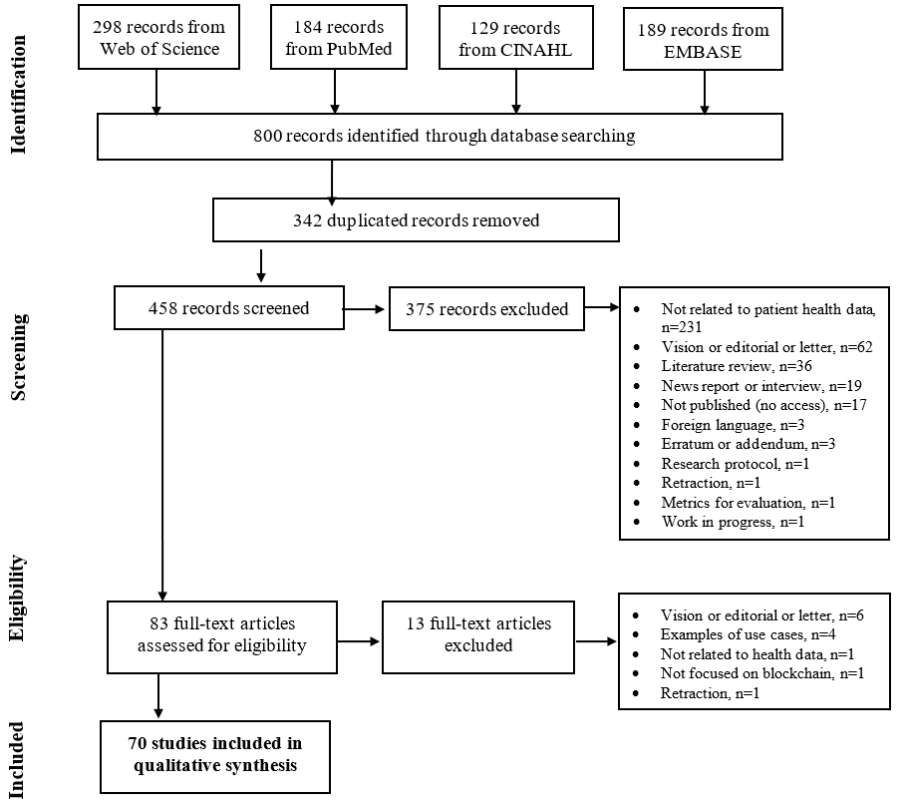
Study identification and selection process. CINAHL: Cumulative Index of Nursing and Allied Health Literature; EMBASE: Excerpta Medica dataBASE.

Two reviewers used Rayyan, a web app for systematic reviews, to independently review all titles and abstracts. A total of 342 duplicates were identified and removed, resulting in 458 papers being selected for full review. The reviewers disagreed on 29 papers, resulting in an inter-rater reliability of 93.7% (429/458). All disagreements were resolved during a consensus meeting. After this assessment, 83 out of the 458 studies remained for analysis. Most of the excluded papers (n=231) were not related to patients’ health data. For example, some studies focused on the application of blockchain in supply chains or cryptocurrency. We labeled 62 excluded papers pertaining to speculations about future blockchain technology projects for the health industry as visionary/editorial/commentary papers. Focusing primarily on peer-reviewed publications, we also excluded news reports, literature reviews, working papers, and research protocols. After screening for full-text eligibility, we identified 70 studies for the final review. Figure 1 presents our study identification and selection process.

We evaluated each paper to determine the following: (1) the types of blockchain-based HIT applications that researchers focused on including electronic health records (EHRs), personal health records (PHR), health information exchange (HIE), and telemedicine; (2) benefits of using blockchain for HIT; (3) health care activities that would benefit from their use, including patient engagement and empowerment, medical/clinical research, and provider access and use; and (4) the barriers and challenges associated with the implementation and maintenance of blockchain-based HIT.

The next stage of analysis was to further characterize the studies based on 4 categories that represent the issues that blockchain technology addressed in each paper: security, privacy, interoperability, and health care outcomes. These categories were determined a priori based on prior research that identified these as major challenges experienced during HIT implementation and use [7-9]. As the analysis proceeded, additional subcategories emerged. We also merged data access, security, and privacy into a single category. At each stage of the review, the reviewers resolved all disagreements by discussion and reached a consensus. Further details can be found in Multimedia Appendix 2.

In the next section, we report on the results of this analysis.

## Results

### How Blockchain Addresses HIT’s Challenges

In this section, we examine the current HIT challenges that blockchain addresses.

Health care data have special properties. The content must be true and complete, and the data must be traceable and resistant to alteration, forgery, or deletion. Due to legal requirements to preserve patient privacy and secure protected health information, health care data must also be safe and anonymous when stored. Health care data contain sensitive personal information, and thus, prevention mechanisms must be established to prevent unauthorized staff from obtaining and extracting information. To facilitate medical or clinical research, health care data must be made accessible while hiding the identity of the patients to whom it belongs. In addition, the data should be encrypted so that once the data are stolen, they cannot be understood without decryption.

HIT faces challenges with respect to ensuring data security, privacy, and integrity. Sharing health care data is challenging because patient data are often stored in disparate systems and achieving interoperability presents challenges such as connecting heterogeneous systems securely, restricting access by unauthorized parties, and maintaining data integrity. Poor health care data quality adversely affects the quality of health care outcomes.

Prior research shows that blockchain can help to overcome the aforementioned challenges. We categorize this research into 3 groups to represent the main HIT challenges that blockchain can address: data security and privacy, interoperability, and health care quality outcomes (Table 1).

Most of the research reviewed focused on blockchain’s use to strengthen HIT security or patients’ privacy during health data exchange or access; 53% (37/70) papers focused on addressing patients’ lack of control over the privacy and security of their data [10-46], and 40% (28/70) papers addressed blockchain’s ability to prevent data tampering [10,12,21,25,33,34, 37,38,40-44,46-60]. Data breaches were addressed in 37% (26/70) papers [15,18,19,22,24,29,31,32,34,36-38,43,44, 54,56-66], 9% (6/70) papers mentioned malicious attacks (eg, impersonation) that blockchain could potentially resolve [17,34,44,56,60,67], and 4% (3/70) papers focused on how blockchain can preserve patients’ anonymity while third parties accessed their health and medical records for activities such as medical research [41,42,68].

The research studies also investigated how blockchain addresses HIT interoperability issues. Interoperability is the ability of different information systems, devices, and applications (*systems*) to access, exchange, integrate, and cooperatively use data in a coordinated manner, within and across organizational, regional, and national boundaries, to provide timely and seamless portability of information [69]. Given that HIT infrastructure might vary by hospital, department, and other structural divisions, HIT incompatibility may arise when data transfer is attempted across these systems, as in the case of HIE; 9% (6/70) papers evaluated blockchain’s use for resolving the poor incompatibility of existing HIT [21,22,26,27,30,55], and 4% (3/70) papers focused on how blockchain can address the poor integration of large volumes of data from different sources [26,37,61]. One challenge to interoperability is the waiting time for data to be updated, affecting the temporal facet of interoperability and real-time access. The lack of real-time access in interoperable systems was discussed in 6% (4/70) papers [13,40,70,71]. The inconsistency of data structures across heterogenous systems makes it difficult to ensure data integrity when data transfer is attempted across interoperable systems; 23% (16/70) papers indicated that blockchain could enhance data integrity across interoperable systems [16-18,33,35-37, 40,41,44,49,64,66,72-74]. Furthermore, data transparency (which refers to the accessibility to data despite its location, data credibility, and data accuracy) across interoperable systems was discussed in 14% (10/70) papers [14,33,40,47, 52,59,70,72,75,76].

The third category covers HIT’s alignment with the health organizations’ goals to achieve specific health care outcomes such as health care quality and system satisfaction; 10% (7/70) papers focused on addressing the inefficiency of current health systems, such as lengthy processing times and the inability to simultaneously process large data volumes [16,24,28,37,55,67,77]. With regard to the quality of patient care, quite often, missing or incorrect health data lead to repetitive lab tests or diagnostic errors, which can be detrimental to patients’ health; 9% (6/70) papers discussed how blockchain can reduce misdiagnosis and overtreatment [27,32,35,40,78,79]. Data dredging may occur in clinical trials where researchers may alter or omit data to achieve a statistically significant result in their experiments; 4% (3/70) papers addressed data dredging [32,52,70]. Another issue is the lack of trust between stakeholders (patients, providers, and researchers) because of stakeholders’ capability to modify and edit health data, which may lead to errors. A total of 4% (3/70) papers examined how blockchain could facilitate the immutability and traceability of health records updates [32,61,80]. The high cost of maintenance of the current health systems was mentioned in 3% (2/70) papers [37,73]. Blockchain technology could potentially reduce maintenance costs by increasing the efficiency and speed of health care data management.

**Table 1:** Health information technology challenges that blockchain addresses.

Challenge		Frequency (N=70), n
**Data access, security, and privacy**		
	Patient lack of control over data	37
	Data tampering	28
	Data breaches	26
	Other malicious attacks (eg, impersonation)	6
	Nonanonymous access to records	3
**Interoperability**		
	Lack of data integrity	16
	Lack of data transparency	10
	Poor compatibility with existing electronic health structures	6
	Lack of real-time access to results	4
	Poor integration of large volumes of data	3
**Health care outcomes**		
	Low efficiency	7
	Misdiagnosis/overtreatment	6
	Data dredging	3
	Lack of trust between stakeholders	4
	High cost of maintenance and data management	2

In the next section, we discuss how blockchain addresses the current challenges surrounding data security and privacy, interoperability, and health care outcomes across a broad spectrum of health care applications and activities.

### Blockchain HIT Applications and Health Care Activities Impacted

Table 2 illustrates the different types of blockchain HITs that researchers focused on. Blockchain HIT research focuses predominantly on improving medical information management systems (MIMS) such as EHRs, electronic medical records (EMRs), and PHRs.

**Table 2:** Blockchain health information technology applications.

Blockchain health information technology	Frequency (N=70), n
Medical information management system	36
Personal health records	7
Clinical trial or health research platform	6
Health information exchange	6
Remote patient monitoring	5
Mobile health	3
Medical image sharing platform	2
Telemedicine platform	2
Predictive or classification modelling	2
Pervasive social network	1
Data preservation systems	1

**Figure 2:**
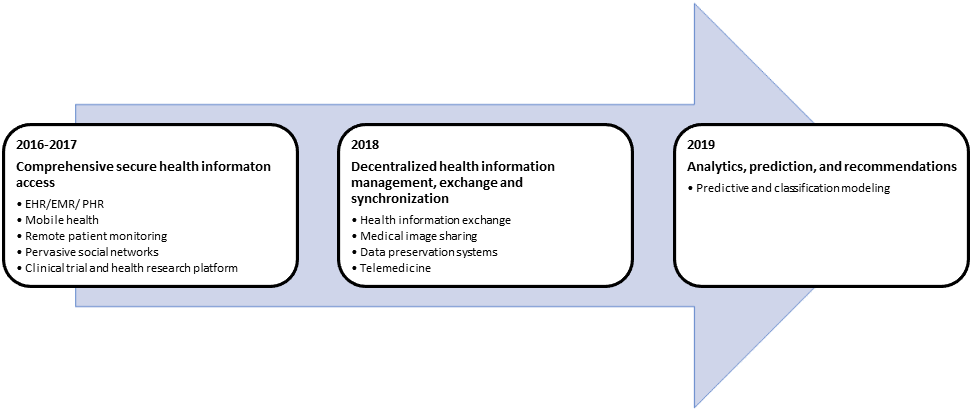
Blockchain health information technology research evolution over time by date of first mention.

We analyzed the blockchain HIT research by the year of first mention (Figure 2). In 2016-2017, researchers worked on providing comprehensive and secure real-time access to patient data in HIT applications such as EHR, EMR, PHR, mobile health (mHealth), remote patient monitoring (RPM), pervasive social networks (PSNs), and clinical trial systems. Research during this period focused on increasing data security and privacy and leveraging blockchain’s unique properties of decentralization, immutability, anonymization, and transaction synchronization to provide a single view of patient data. In 2018, blockchain HIT research expanded to include improving health information management, exchange, and synchronization across decentralized nodes in telemedicine, medical image sharing, data preservation systems (DPSs), and HIE. In 2019, blockchain HIT research expanded to leveraging blockchain’s decentralization properties to eliminate a single point of control while carrying out predictive analytics and classification modeling across multiple institutions.

**Figure 3:**
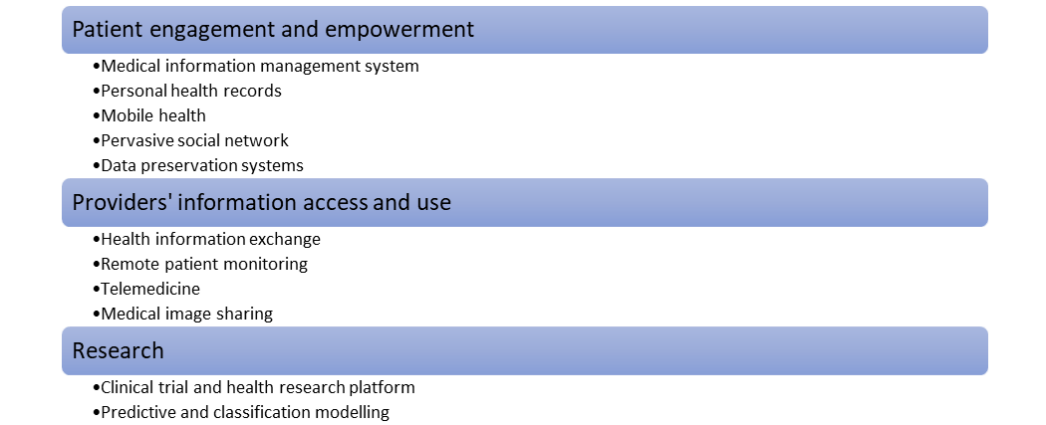
Health care activities that blockchain health information technology impacts.

Blockchain HIT improves patient engagement and empowerment, research quality and processes, and provider’s information access and use (Figure 3). We describe how blockchain can improve each of these areas in further detail in the next section.

#### Patient Engagement and Empowerment

We define patient engagement as the act of patient and provider working together to improve the patient’s health [81]. Patient engagement may result in patient empowerment. Patient empowerment refers to a process through which patients gain greater control over decisions and actions that affect their health [82]. Patient engagement and empowerment promote shared decision making about the patient’s care by both the providers and the patient and patient-centered care that is highly responsive to individual patients’ preferences, needs, and values [83].

Patient engagement and empowerment may benefit from 6 types of blockchain HIT: MIMS, PHRs, mHealth, telemedicine, PSNs, and DPSs.

##### Medical Information Management Systems

MIMS refer to EHRs and EMRs, which store and manage patients’ health information. The EHR is a computerized and standardized information model with information relevant to the health and wellness of an individual, enabling integration among multiple health care providers [30]. The EMR differs from the EHR as it focuses on the internal medical domain of health organizations and is not integrated between health care providers. Blockchain’s use for EHRs, EMRs, and other record systems in which records are originated and controlled by providers was discussed in 51% (36/70) papers [11,14-21,27,29, 30,33,35-37,40,42-44,46,49,53,55,59,60,63,67,68,72-74,76-78,84]. Using blockchain to integrate various health record systems (EHR, EMR, PHR, etc), mHealth, and telemedicine platforms can contribute to patient empowerment and patient engagement by providing a synchronized view of patient information to patients and health care providers, to support patient decision making and control.

It is challenging to keep track of all data and ensure its immutability in the context of complex medical cases that require multiple diverse activities, patients’ visits, and providers’ treatments [17]. Blockchain-based MIMS facilitate the creation of immutable records with traceable transactions that cannot be changed over time. Blockchain can provide patients and providers with more secure and private access to the data and allow them to better collaborate in complex medical situations.

Blockchain technology facilitates smart contracts, which is a computer protocol intended to digitally facilitate, verify, or enforce the negotiation or performance of a contract between two parties without a third party’s involvement. Smart contracts can facilitate formalized contracts to streamline patient consent for treatment, patient authorization for access to and release of PHI, and the authentication and verification of those seeking access to patient data. Patient privacy is also enhanced as blockchain can allow patients to authorize access to their records while remaining anonymous, for example, for research purposes. Patients can also publish medical information to the blockchain anonymously, which is also useful for sharing information with third parties and for research purposes. Blockchain’s properties, including its cryptographic techniques and immutability and autonomy, enhance user authentication and verification, access control, and HIT security and privacy [18].

In the health care sector, the resilience of health records systems in medical information systems is crucial, given the increasing number of data breaches in health care [85]. Blockchain’s distributed design is more resistant to malicious attacks, as there is no single point of control or failure. Overall, such increased security and privacy may motivate patients to engage in full disclosure of their condition and contribute to patient empowerment by facilitating better provider feedback, counseling, and patient decision making.

##### PHRs

PHR refer to the representation of information regarding an individual’s health, including wellness, development, and welfare, which the patient owns and can share with third parties [30]. PHR is oriented to the patient but can be integrated with the EHR. Blockchain’s use for PHR was discussed in 10% (7/70) papers [13,23,25,26,30,64,78]. PHR’s main challenges include poor interoperability with other systems, large data volumes, outdated data, duplication, lack of standardization, and fragmentation. Due to its distributed design, a permissioned blockchain is well positioned to address these challenges by providing a distributed ledger where transactions can be recorded by multiple, authenticated parties. Due to its decentralized design, blockchain-based PHR can provide patients with a unified view of their scattered health records by interconnecting scattered patient data across several health care organizations. Furthermore, as the PHR is updated with the patient’s medical conditions and services received, health providers can become aware of changes in their patients’ conditions and alter and modify treatment plans, even though updates may occur elsewhere. These features can improve the communication effectiveness between a provider and a patient, providing the patient with the information needed to take charge of their health care decision making. In terms of patient engagement and empowerment, blockchain-based PHR can provide patients with control over their health data and facilitate providers’ real-time updates of patient data.

##### mHealth and Telemedicine

mHealth, telemedicine platforms, DPSs, and PSNs can also contribute to patient engagement and empowerment. A total of 4% (3/70) papers focusing on mHealth indicated that blockchain’s distributed design can prevent data tampering and provide a single point of control [22,31,51]. Data tampering may occur during a malicious attack and lead to a loss of data reliability within the mHealth application [51]. Tamperproof systems are critical in scenarios where treatment is automatically administered to patients without human intervention, based on the data the mHealth application collected. Tamperproof systems are also essential to ensure that medical decision making is based on accurate information to prevent harm to patients. Blockchain enables tamper-resistant systems by maintaining a constantly growing list of transactional records divided into blocks and uses consensus algorithms to allow multiple parties to agree on a common state. Due to its distributed design, blockchain is well suited to mHealth applications as it allows for frequent updates.

A total of 3% (2/70) papers [48,71] mentioned blockchain-based tele-dermatology and other telecare platforms. Blockchain enhances telemedicine platforms by (1) anonymizing patients’ information and access; (2) decentralizing control and allowing patients to manage their data and consultations independently; (3) providing a secure global EHR to share and exchange data for research, teaching, or clinical work; (4) limiting health care fraud by using smart contracts to automate invoice processing; (5) eliminating mediators and reducing administrative costs; and (6) fundraising through ICOs (eg, DocCoin, PointNurse, and Medical Chain). In the case of DermaNet, the tele-dermatology platform, blockchain shortens the virtual distance between patients and providers by efficiently sharing information about changes in dermatological disease, improving quality of care, and allowing actors to trust each other [44]. Building trust between health care providers and patients supports patient engagement and empowerment.

##### DPSs and PSNs

DPSs notarize *data to provide legal evidence for medical disputes and medical negligence* [58]; 1% (1/70) of papers addressed how blockchain can enhance DPSs [58].

Current DPSs may be unreliable as persons can tamper with the data, reducing the accuracy of patient information. If a third-party notarized company is contracted to provide preservation services, personal information may be leaked, and it is difficult to guarantee the reliability and availability of DPSs [66].

Blockchain provides an immutable, distributed transaction ledger that reduces the abovementioned challenges associated with DPSs. Blockchain-based DPSs provide a reliable and secure data storage solution that prevents data tampering and allows transactions to be synchronized. Decentralization makes the DPS more secure, as attackers are not able to attack all nodes simultaneously. Information security and data integrity are strengthened as blockchain’s synchronization and consensus algorithms ensure that all copies of the data are the same at every node. The DPS is capable of storing patient data anonymously, thus preserving patient privacy in the event of tampering or breach.

A total of 1% (1/70) of papers focused on the creation of a secure system for PSN-based health care systems [54]. PSN-based health care systems enable users to share health data that medical sensors collect, for disease monitoring and control and remote medical care. Due to its distributed design, blockchain reinforces the synchronization of transactions and operations in such systems, increasing health information’s security, reliability, and accuracy. These features contribute to patients’ confidence in the security and privacy of their data and promote health care engagement and empowerment.

#### Health Care Provider’s Health Information Access and Use

##### HIEs, RPM Systems, and Medical Image Sharing Platforms

Blockchain-based HITs such as HIE, RPM systems that leverage Internet of Things (IoT) technologies, and medical image sharing systems provide benefits to health care providers by improving health information access and use.

HIE refers to the process of electronic transfer of patient health information and medical data among health care providers and institutions so as to provide health care providers with better information for diagnosis and patient treatment. The potential benefits of HIE include decreasing rates of patient readmission, avoiding medication errors, improving diagnoses, and decreasing duplicate testing [20]. However, HIE systems suffer from challenges such as threats to patient privacy, information security threats and vulnerabilities, poor integration of disparate data sources, and dependency on centralized data storage [16]. Furthermore, most HIE are designed for health care providers, and patients cannot access their data in the HIE when they visit hospitals outside their home systems; 9% (6/70) papers discussed how blockchain could improve HIE systems to facilitate the sharing of health records between providers, patients, and hospitals [16,32,39,50,57,79]. These papers suggest that blockchain’s features of decentralized transaction validation, data provenance insurance, data sharing, and data integration may resolve HIE’s challenges. For example, as blockchain technology utilizes distributed databases to store all transactions, it is possible to design a permissioned blockchain system to connect multiple EHR databases from different clinical sites to perform information exchange [20]. The private chain allows only authorized users to join the system, and smart contracts regulate all transactions. These blockchain-enabled smart contracts ensure data provenance and immutability, decentralization, restricted access to and anonymity of patients’ records, and other benefits.

RPM enables health care providers to use sensor data to monitor patients outside of the clinical setting [12,41,62,66,75]; 7% (5/70) papers explored the use of blockchain in RPM systems. As the RPM realm expands, concerns about efficient and secure transmission of medical data arise as data come from different sensors, are a lucrative target for hackers, and must conform to health care data security and privacy regulations. Furthermore, patient treatment events initiated by IoT must be securely logged to show the patient’s treatment and a record of who permitted it, to protect the integrity of the patient’s care and maintain an accurate timeline of events.

These papers proposed the design of blockchain-based IoT technology to build a network architecture to better manage data from remote sensors to address these challenges. For example, smart contracts could support real-time patient monitoring and medical interventions by sending notifications to patients and medical practitioners while maintaining a secure record of who initiated these activities [77]. Blockchain-based IoT systems could also allow patients to maintain anonymity, create a permanent digital trace of patients’ health records, and prevent a single point of control or failure through a highly decentralized system structure. These features would allow providers to securely access their patients’ data and better control their patients’ conditions.

A total of 3% (2/70) of papers focused on the development of blockchain-based cross-domain medical image sharing platforms [24,65]. In the past, digital images between providers were predominantly shared through a physical copy (eg, a CD) [24]. To address issues associated with digital image transfer, the Radiological Society of North America developed the Image Sharing Network that allows digital image transfer through a third-party clearinghouse. This raised new concerns regarding data storage centralization and intermediaries’ involvement in the medical image exchange process [24]. Blockchain solves these concerns by decentralizing the entire system by removing the need for a central intermediary, creating immutable records that ease communication between providers, and eliminating third-party access to protected health information. This approach satisfies many criteria of an interoperable health system and is generalizable to other contexts [56].

#### Health Care Research

##### Clinical Trial Management and Health Care Research

When conducting medical research, data records are usually widely accessible, but the patients to whom they refer are anonymous [84]. Blockchain improves health care research practices and supports patient data anonymization during the research process through sophisticated cryptographic techniques. A total of 9% (6/70) of papers focused on creating blockchain-based systems capable of improving clinical trial management and enhancing patients’ trust in health care research, impacting activities such as collecting, storing, and tracking patients’ informed consent; improving data integrity; and sharing clinical data between providers [10,16,52,61,70,80]. This is particularly relevant to clinical investigator–related deficiencies, as the US Food and Drug Administration reports that about 10% of clinical trials suffer from consent collection issues such as unapproved forms and outdated consent documents [10]. In BlockTrial [52], consent algorithms in the blockchain system can enable clinical research stakeholders to share and update patient consent forms and retrieve relevant data. In addition, the system can empower patients to become more active and fully informed research partners [52]. Blockchain can also ensure data accuracy in clinical trials without confirmation by a third-party contract research agency, thus reducing the cost of clinical trials [63].

In the health care analytics domain, blockchain was used in privacy-preserving predictive modeling [47] and a cloud-based health resource–sharing model used for breast tumor diagnosis [79]. In the case of blockchain-based privacy-preserving modeling, the removal of the *server* role eliminated a single point of control [47]. This single point of control poses various security risks, as multiple institutions that want to create a generalizable model on health or genomic data would have to rely on a single party.

### Blockchain HIT Implementation Challenges

As shown in Table 3, despite blockchain’s multiple advantages for health care use, studies indicated various downsides. These limitations point to open areas for research; 16% (11/70) papers revealed that blockchain technologies have the capability of enhancing security and privacy, yet have security and privacy vulnerabilities and weak access control mechanisms [23,24,32-34,41,57,58,70,75,79]. For example, blockchain-based structural health monitoring systems do not have mechanisms to guarantee the security of the data placed on the blockchain, although it is expected that the blockchain would enhance the security of the overall system [75]. Furthermore, research has shown that although it is almost impossible to alter and modify blockchain records in an EMR system, it is possible to tamper with and hack smart contracts [40].

Researchers have discussed high computing power and implementation costs in 16% (11/70) papers [12,23,25,29,47,48,52,57,61,76,79]. Prior research shows that blockchain-based EHRs consume significant computational power and take a large amount of time to execute tasks [29]. Blockchain-based clinical trial platforms that utilize Ethereum blockchain technology are also costly [52].

Researchers raised concerns about lengthy response and transaction processing times in 7% (5/70) papers [12,32,36,41,57]. For example, blockchain RPM systems rely on sensor-based data that must be accumulated, acted upon, and added to the blockchain, potentially introducing delays [41].

The potential resistance to blockchain adoption by patients and providers was raised in 7% (5/70) papers [11,24,32,48,70]. Public perceptions of blockchain technology might stand in the way of the successful implementation of blockchain-based EHRs because of blockchain’s nascency and association with the negative use of blockchain-based technology such as cryptocurrencies within black markets [11].

Although researchers have indicated that blockchain-based electronic health (eHealth) structures may resolve the incompatibility of existing eHealth structures [21,22,26,27,30,55], new blockchain health systems may be incompatible with health care legacy systems [13,48,52,61,76]. Therefore, more research is needed to investigate approaches to address the interoperability between legacy and blockchain systems.

A total of 6% (4/70) of papers pointed out issues associated with blockchain’s consensus algorithms [33,36,51,75]. For example, in small networks with a limited number of peers, the Practical Byzantine Fault Tolerance algorithm, which is designed to prevent catastrophic system failures due to malicious nodes, can be disabled if more than a third of the peers are offline at the same time. Therefore, it is important to increase the number of peers to prevent malicious peers from occupying the entire system.

Linking participants to their digital identity is a predominant concern [10,13,25]. Guaranteeing the blockchain informant’s identity and authenticity is not foolproof, whether the informant is a patient, physician, or a sensor connected to a patient. Although blockchain technology helps prevent data block fraud, it is challenging to ensure that only authentic informants can access the health records and to prevent attacks on the blockchain [55]. Although blockchain demonstrates the potential to preserve the privacy of patients, further testing for security, privacy, and user authentication is needed.

Another constraint is data storage limitations, as discussed in 4% (3/70) studies [23,25,60]. Stakeholders are constrained by the amount of data that they can store in the blockchain. Furthermore, legislation such as Article 17 Right to Erasure, in Europe’s General Data Protection Regulation (GDPR), gives citizens the right to request the modification and deletion of personal data, which is difficult, given the permanency of data recorded on the blockchain [19].

**Table 3:** Blockchain health information technology barriers and challenges.

Barrier or challenge	Frequency (N=70), n
No guarantee of security or privacy	11
Computing power and implementation costs	11
Users’ resistance to a new technology	5
Long response time	5
Integration of existing electronic health structures into blockchain	5
Issues (eg, lack of efficiency) associated with consensus algorithms	4
Linking participants to their digital identity	3
Data storage limitation	3
Not mentioned	44

## Discussion

### Principal Findings

We presented the current state of research on blockchain technologies in patient care. We identified 3 main categories of research: data privacy and security, interoperability, and health care outcomes. We highlighted the health care applications that leverage blockchain technology. These health care applications include MIMS (EHR and EMR), PHRs, mHealth and telemedicine, DPSs and social networks, HIEs, remote monitoring systems, and medical research systems. These applications may improve patient engagement and empowerment, improve health care providers’ access to information, and enhance health care information use for medical research. However, several challenges and implementation barriers exist, such as security and privacy vulnerabilities, user resistance, high computational power and implementation costs, inefficient consensus algorithms, and challenges integrating blockchain with existing HIT. Despite blockchain’s disruptive potential, it is important that blockchain’s limitations are further examined and understood and available alternatives considered before embarking upon any large-scale blockchain implementation.

### Future Directions

Following are some of the challenges that have received the least attention in the literature. Therefore, researchers should focus on investigating the following research questions.

1. How can blockchain's interoperability and compatibility with existing HIT infrastructures be improved?
2. How can blockchains storage limitations be addressed?
3. How can blockchain adoption be promoted?
4. What blockchain HIT research should be pursued to disrupt health care?

### Improving Blockchain’s Interoperability and Compatibility With Existing HIT Infrastructure

Organizations need to understand how to connect their HIT blockchain to compatible blockchains, noncompatible blockchains, or nonblockchain platforms. Research should focus on ascertaining effective integration governance models and new interoperability and data standards and exploring performance optimization approaches. Researchers should also explore the feasibility of integration approaches for health care systems, such as cross-authentication (for compatible blockchains), oracles (which transfer external data to the blockchain for on-chain use), or application programing interfaces (for incompatible blockchains). The feasibility of translators that use open standards such as Health Level 7 and Fast Healthcare Interoperability Resources and *open* EHR or an equivalent ontology to connect proprietary systems to blockchain HIT is an open area of research [13]. Blockchain silos that different stakeholders create present complexity and interoperability challenges that should be investigated. An understanding of the legal and regulatory implications of blockchain interoperability is needed, as security and privacy concerns are pertinent issues.

### Addressing Blockchain Storage Limitations

HIT researchers are becoming aware of the technical limitations regarding data storage on the blockchain, and researchers are beginning to explore alternative approaches to conform with the GDPR. Recommendations include recording metadata such as addresses, hash values, and time stamps on the blockchain while storing PHI off-chain elsewhere, such as in the cloud or on hospital servers [23]. However, splitting data storage may degrade system performance. Future research should determine feasible ways to optimize the cost, performance, and efficiency of implementations that split data storage on and off the blockchain.

### Promoting Adoption of Blockchain

The public’s negative perception of blockchain applications, such as cryptocurrencies, contributes to user resistance [11]. Lessons learned from the financial technology domain show that users are motivated to adopt cryptocurrencies when these applications are aligned with users’ value systems [86]. To support this perspective, nascent HIT research shows that patients hold a favorable attitude toward the implementation of blockchain-based HIE mechanisms for privacy protection, coordination, and information exchange purposes [32]. User resistance may also arise because stakeholders do not understand blockchain technology. Researchers can explore how industry consortia of influential health care players working together to educate stakeholders and illustrate proof of concept may increase network effects and spur adoption. Further research could also explore what incentivizes stakeholders to work together to adopt blockchain and to find common solutions. The role of the blockchain HIT startup ecosystem in stimulating user adoption is also a relevant area of research. Researchers can also explore how to lift adoption barriers presented by legal and regulatory constraints caused by legislation such as the Health Insurance Portability and Accountability Act of 1996 (HIPAA) and the GDPR, which did not contemplate the unique ways in which blockchain technology handles data privacy and security. Considerations of what should be stored on and off chains are important for HIPAA and GDPR compliance and adoption.

### Pursuing Research Opportunities for Disruption of Health Care

HIT researchers are currently focused on improving health care applications, but there is room for researchers to explore blockchain HIT’s disruptive potential. Most HIT research has focused on the use of smart contracts in HIT applications. It is worth noting that smart contracts are the building blocks of decentralized autonomous organizations (DAOs) and decentralized applications and services (D-APPs). DAOs and D-APPs have the potential to facilitate novel DAOs to disrupt patient care in areas such as organ and blood donations, electronic prescriptions, laboratories, and other diagnostic services.

### Limitations

This review did not focus on the use of blockchain in the health industry outside of patient care, such as in the health insurance marketplace, pharmaceutical industry supply chain, or the health care provider credentialing process where blockchain is being proposed to reinvent these industries. The COVID-19 pandemic has exposed many high-value use cases where blockchain-enabled technology could address evolving societal needs and support accelerated responses to disruptions, such as supply chain management to connect health care organizations to trusted sources for necessary medical supplies, tracking and monitoring drugs’ origin and journey from pharmacy to patient administration, and verifying credentials of clinicians and health care professionals volunteering at hospitals to alleviate workforce demands. A further review could explore blockchain research in these areas as part of the pandemic response.

### Comparison With Prior Work

Prior reviews included conceptual papers, industry reports, and empirical research and primarily focused on identifying use cases and associated challenges [1-3,6,87-89]. To ascertain the current state of research, this review only focused on empirical research on the use of blockchain for patient care. Prior reviews included studies up to 2018 [1-3,6,87-89]. In this review, 58 of the 70 included empirical research studies were published in 2018 and 2019. We further distinguish this review by describing how blockchain HIT research focused on patient care evolved over time, from 2016 to 2019.

We build upon a systematic review of the blockchain HIT literature up to 2018 conducted by Agbo et al [6], which differs in its focus, inclusion criteria, results, and research agenda. Specifically, Agbo et al [6] identified 4 blockchain applications used for patient care: EMRs (which we expanded to include EHRs and PHRs); research; RPM; and health analytics, which we further broke down into predictive analytics and classification modeling. This review updates the blockchain HIT applications reported in the study by Agbo et al [6] by identifying 5 additional discrete categories of blockchain HIT research focused on patient care: HIEs, medical image sharing, mHealth and telemedicine, PSNs, and DPSs. Agbo et al [6] identified 5 challenges when implementing blockchain HIT applications: interoperability, security and privacy, scalability, speed, and patient engagement. Challenges found in our review but not included in the study by Agbo et al [6] included computing power and implementation costs, user resistance during implementation, consensus algorithm inefficiencies, difficulties in linking patients to their digital identity, and data storage limitations. To validate our search terms, we performed an alternative search of the literature using the search terms provided by Agbo et al [6]. However, the search terms we developed provided more relevant results for our purposes than the search terms by Agbo et al [6].

### Conclusions

We have presented the current state of research on the use of blockchain technologies with a focus on patient care. Although blockchain presents significant potential for disrupting health care, most ideas are in their infancy. With 51% of the research focused on medical information systems such as EHR and EMR and 53% of the research focused on data security and privacy issues, this review shows that HIT research is primarily focused on the use of blockchain technologies to address the current challenges HIT faces. Future research can focus on how blockchain can disrupt patient care and help overcome the challenges in health care delivery post-COVID-19 by creating new decentralized organizations, applications, and services.

## Appendix

Multimedia Appendix 1 Preferred Reporting Items for Systematic Reviews and Meta-Analyses checklist.

Multimedia Appendix 2 Summary of the included studies.

## Abbreviations

CINAHL: Cumulative Index of Nursing and Allied Health Literature
DAO: decentralized autonomous organizations
D-APPs: decentralized applications and services
DPS: data preservation system
eHealth: electronic health
EHR: electronic health records
EMBASE: Excerpta Medica dataBASE
EMR: electronic medical records
GDPR: General Data Protection Regulation
HIE: health information exchange
HIPAA: Health Insurance Portability and Accountability Act of 1996
HIT: health information technology
ICO: initial coin offerings
IoT: Internet of Things
mHealth: mobile health
PHI: personal health information
PHR: personal health records
PSN: pervasive social networks
RPM: remote patient monitoring
