# Supplementary material for: The Current State of Research, Challenges, and Future Research Directions of Blockchain Technology in Patient Care: Systematic Review
Source: J Med Internet Res. 2020 Jul 20;22(7):e18619. doi: 10.2196/18619 (PMC7399962; doi:10.2196/18619)
Supplement: Multimedia Appendix 2 [file jmir_v22i7e18619_app2.docx]

## Multimedia Appendix 2: Summary of included studies

| **Citation** | **Purpose** | **HIT** | **Issues Addressed** |
| --- | --- | --- | --- |
| Beinke et al. (2019) | The authors propose a blockchain-based EHR system. | EHR | - Lack of patients’ control over their data - Data tampering |
| Benchoufi et al. (2017) | The authors propose to create a system that allows the collection of patients’ informed consent, storing and tracking the consent in a secure way, and enabling the sharing of this information. | Clinical trial platform | - Lack of patients' control over their data - Data tampering |
| Cunningham & Ainsworth (2017) | The authors propose a blockchain system that enables personalized control of third-party access to EHR. | EHR | - Lack of patients' control over their data |
| Dey et al. (2017) | The authors propose a blockchain-based IoT model through which bio-sensor measures and real-time medical patient data are collected. | RPM | - Lack of patients' control over their data - Data tampering |
| Jo et al. (2018) | The authors propose blockchain-IoT-based network for secure and transparent data sharing in structural health monitoring. | RPM | - Lack of data transparency |
| Kuo et al. (2019) | The authors propose a blockchain-based framework for decentralized privacy-preserving predictive modeling. | Predictive modelling platform | - Lack of data transparency - Data tampering |
| Lo et al. (2019) | The authors propose a blockchain-based system for medical referral between facilities. | HIE | - Lack of patients' control over their data - Lack of trust - Lack of efficiency |
| Mannaro et al. (2018) | The authors propose a blockchain-based approach to enhance functionalities of a teledermatology platform. | Teledermatology platform | - Data tampering |
| Roehrs et al. (2017) | The authors propose a blockchain-based PHR model for patients and providers to access patients' data. | PHR | - Lack of patients' control over their data - Lack of real-time access to results |
| Xia et al. (2017) | The authors propose a blockchain-based a system that addresses the issue of medical data sharing among medical big data custodians in a trust-less environment. | EMR | - Lack of patients' control over their data - Data transparency |
| Xia et al. (2017) | The authors propose a blockchain-based data sharing framework that addresses the access control challenges associated with sensitive data stored in the cloud using immutability and built-in autonomy properties of the blockchain. | EMR | - Lack of patients' control over their data - Data breaches |
| Zhang et al. (2018) | The authors propose the structure and functionality of a blockchain-based architecture for sharing clinical data between distributed providers. | Clinical trials platform | - Data breaches - Lack of trust between stakeholders - Poor integration of large volumes of data |
| Zhao et al. (2017) | The authors design a lightweight backup and efficient recovery scheme for keys of health blockchain. | Remote patient monitoring (RPM) | - Data breaches |
| Zhuang et al. (2018) | The authors implemented a private blockchain system to simulate scenarios in HIE. | clinical trials platform, HIE, EHR | - Lack of patients' control over their data - Lack of data integrity - Inefficiency |
| Alexaki et al. (2018) | The authors present a conceptual medical record access and sharing mechanism for a system operating within regulated health care jurisdiction. | EHR | - Lack of patients' control over their data - Malicious attacks - Lack of data integrity |
| Cao et al. (2019) | The authors propose a cloud-assisted blockchain system to protect EHR from illegal modifications. | EHR | - Data tampering - Lack of data integrity |
| Castaldo & Cinque (2018) | The authors propose a blockchain-based approach for HIE between European countries. | HIE | - Data tampering |
| Dagher et al. (2018) | The authors propose a blockchain-based platform for secure, interoperable, and efficient access to EHR. | EHR | - Data breaches |
| Du et al. (2018) | The authors propose a cloud-assisted and blockchain-based medical information service platform to share patient information in complex network environment. | Medical Information Management System | - Lack of patients' control over their data - Data breaches - Lack of data integrity |
| Fan et al. (2018) | The authors propose a secure blockchain-based system for data sharing among authorized users. | EMR | - Lack of patients' control over their data - Data breaches |
| Guo et al. (2018) | The authors propose an attribute-based sig- nature scheme with multiple authorities for EHR by using blockchain. | EHR | - Lack of patients' control over their data |
| Hyla & Pyjas (2019) | The authors propose a blockchain-based system that ensures transactional transparency in distributed eHealth environments. | EHR | - Lack of data integrity - Data transparency |
| Ichikawa et al. (2017) | The authors proposed a blockchain-based mHealth system that enables trusted and auditable computing via decentralized network. | mHealth | - Data tampering |
| Kaur et al. (2018) | The authors propose a model on the implementation of blockchain network on the cloud environment for storing and managing health care data. | EHR | - Lack of data integrity - High cost of maintenance |
| Kim et al. (2018) | The authors propose a blockchain-based medical questionnaire management system for data sharing. | Medical Information Management System | - Lack of patients' control over their data - Data tampering - Poor compatibility with existing e-health structures |
| Li et al. (2019) | The authors propose a blockchain-based data management system for mobile health care systems. | EMR | - Malicious attacks - Inefficiency |
| Liang et al. (2017) | The authors propose a user-centric blockchain-based health data sharing solution that ensures privacy and enhances the identity management. | mHealth | - Lack of patients' control over their data - Data breaches - Poor compatibility with existing e-health structures |
| Liu et al. (2017) | The authors propose a blockchain-based system for medical data exchange. | EHR | - Lack of data transparency |
| Maslove et al. (2018) | The authors explore the role of blockchain technology in clinical trial management. | Clinical trial platform | - Data tampering - Lack of data transparency - Data dredging |
| Park et al. (2019) | The authors explore the role of blockchain technology in Personal Health Records (PHR) management. | PHR | - Lack of patients' control over their data |
| Patel (2019) | The author developed a framework for blockchain-based cross-domain medical image sharing. | Medical image sharing platform | - Lack of patients' control over their data - Data breaches - Inefficiency |
| Pham et al. (2018) | The authors propose a blockchain-based technology for managing patients' records and medical devices. | PHR | - Data breaches - Lack of data integrity |
| Rahmadika & Rhee (2018) | The authors propose a conceptual blockchain-based framework for managing PHI (personal health information). | PHR | - Lack of patients' control over their data - Data tampering |
| Roehrs et al. (2019) | The authors evaluate the blockchain-based system that they propose in Roehrs et al. (2017). | PHR | - Lack of patients' control over their data - Poor compatibility with existing e-health structures - Poor integration of large volumes of data |
| Sutton et al. (2018) | The authors propose a blockchain-based system to increase trust in health research. | Health research platform | - Lack of trust between stakeholders |
| Silva et al. (2019) | The authors propose a blockchain-based Fog Computing architecture to facilitate the management of medical records. | Medical Information Management System | - Lack of patients' control over their data - Poor compatibility with existing e-health structures - Misdiagnosis/overtreatment; |
| Talukder et al. (2018) | The authors present a consensus protocol that would solve many data challenges associated with EHR and HIE. | HIE, EHR, EMR, PHR | - Misdiagnosis/overtreatment |
| Tang et al. (2018) | The authors propose a blockchain-based system for medical image sharing with integration of credit score information. | Medical image sharing platform | - Data breaches |
| Tang et al. (2019) | The authors propose an efficient authentication scheme for blockchain-based EHR. | EHR | - Lack of patients' control over their data - Inefficiency |
| Uddin et al. (2018) | The authors propose a blockchain-based end-to-end architecture for continuous patient monitoring that has a patient centric agent (PCA) as its center piece. | Remote patient monitoring (RPM) | - Data breaches - Poor data integrity |
| Vora et al. (2018) | The authors propose a blockchain-based platform for efficient storage and maintenance of EHRs. | EHR | - Lack of patients' control over their data - Data breaches |
| Wang & Song (2018) | The authors propose a blockchain-based EHR to achieve confidentiality, authentication, integrity of medical data, and support fine-grained access control. | EHR | - Data tampering |
| Wang et al. (2018) | The authors propose a blockchain-based parallel health care systems (PHSs). | EHR, PHS | - Lack of patients' control over their data - Poor compatibility with existing e-health structures |
| Wong et al. (2019) | The authors propose a blockchain-based system to make data collected in the clinical trial process immutable, traceable, and potentially more trustworthy | Clinical trial platform | - Lack of data transparency - Lack of real-time access to results |
| Xiao et al. (2018) | The authors propose a blockchain-based cross-organizational medical data sharing framework. | EMR | - Inefficiency |
| Yue et al. (2016) | The authors proposed an App architecture based on blockchain to enable patient to own, control and share their own data. | mHealth | - Lack of patients' control over their data - Data breaches |
| Zhang et al. (2016) | The authors propose a PSN-based health-care system that relies on security protocols. | Pervasive social network (PSN) | - Data breaches - Data tampering |
| Chen et al. (2019) | The authors propose a blockchain based searchable encryption scheme for EHRs. | EHR | - Data tampering - Lack of data integrity - Poor compatibility with existing e-health structures - Inefficiency |
| Esmaeilzadeh & Mirzaei (2019) | The authors aimed at exploring the core value of blockchain technology in the health care industry from health care consumers’ views. | HIE | - Lack of patients' control over their data - Data breaches - Lack of trust between stakeholders - Data dredging - Misdiagnosis/overtreatment - Lack of real-time access to results |
| Hang et al. (2019) | The authors propose a blockchain-based platform to secure the EMR management | EMR | - Lack of patients' control over their data - Data tampering - Lack of data integrity - Lack of data transparency |
| Kubendiran et al (2019) | The authors propose a blockchain-based approach to complement e-health systems. | E-health systems | - Lack of data integrity |
| Motohashi et al. (2019) | The authors designed and validated an mHealth system that enables the compatibility of the security and scalability of the medical data using blockchain technology | mHealth | - Malicious attacks - Data tampering - Data breaches - Lack of patients' control over their data |
| Thwin et al. (2019) | The authors aim to propose a blockchain-based PHR system. | PHR | - Data tampering - Data breaches - Malicious attacks - Inefficiency |
| Tian et al. (2019) | This paper proposes to establish a shared key that could be reconstructed by the legitimate parties before the process of diagnosis and treatment begins. | Medical data platform | - Lack of patients' control over their data - Lack of data integrity - Misdiagnosis/overtreatment |
| Yang et al. (2019) | This study utilizes the transparency, security, and efficiency of blockchain technology to establish a collaborative medical decision-making scheme. This | EMR | - Data breaching - Lack of patients' control over their data - Lack of data integrity |
| Zheng et al. (2019) | This study aimed to develop a health-related data sharing system by integrating IoT and DLT to enable secure, fee-less, tamper-resistant, highly-scalable, and granularly-controllable health data exchange, as well as build a prototype and conduct experiments to verify the feasibility of the proposed solution. | Health data sharing system | - Data tampering - Data breaches |
| Zhou et al. (2019) | The authors proposed a model called Med-PPPHIS, which consists of a permission-less blockchain and a permissioned blockchain, named Med-DLattice, to serve the management of user’s personal health information and form a chained protection mechanism for medical data. | Medical data management system | - Lack of control of access to data - Data breaches - Data tampering - Poor integration of large volumes of data - Inefficiency - High cost of maintenance |
| Badr et al. (2018) | The authors propose a novel protocol to achieve a perfect privacy preserving for the patient namely Pseudonym Based Encryption with Different Authorities (PBE-DA) by applying the concept of Blockchain on the health care communication entities in an e-health platform | EHR | - Non-anonymous access |
| Chen et al. (2018) | The authors designed a storage scheme to manage personal medical data based on blockchain and cloud storage. |  | - Lack of patients' control over their data - Data breaches - Data tampering |
| Cichosz et al. (2019) | This work aims to present an approach for a blockchain-based platform for sharing health care data. | Diabetes management platform | - Lack of patients' control over their data |
| Dubovitskaya et al. (2019) | The authors propose their perspectives on blockchain based health care data management, in particular, for EMR data sharing between health care providers and for research studies. | EMR | - Lack of patients' control over their data - Data tampering - Data integrity - Lack of real-time access to results - Lack of data transparency - Misdiagnosis/overtreatment |
| Griggs et al. (2018) | The authors propose utilizing blockchain-based smart contracts to facilitate secure analysis and management of medical sensors. | Remote patient monitoring (RPM), EHR; | - Lack of real-time access to result - Data tampering - Non anonymous access to records - Lack of data integrity - Lack of patients' control over their data |
| Kurdi et al. (2019) | This paper addresses data security and trust challenges by proposing HealthyBroker, a novel, trust-building brokering architecture for multiple cloud environments. | E-health systems | - Lack of patients' control over their data - Data tampering - Non-anonymous access to records |
| Hussein et al. (2018) | The authors propose a system supported by a Discrete Wavelet Transform to enhance the overall security, and a Genetic Algorithm technique to optimize the queuing optimization technique as well. | EMR | - Data breaches - Data tampering - Lack of patients' control over their data - Lack of data transparency |
| Ji et al. (2018) | This paper investigates the location sharing based on blockchains for telecare medical information systems. | Telemedicine platform | - Lack of real-time access to results |
| Li et al. (2018) | In this paper, the authors propose a novel blockchain-based data preservation system (DPS) for medical data. | Data preservation system (DPS) | - Data breaches - Data tampering |
| Nguyen et al. (2019) | The authors propose a novel EHRs sharing framework that combines blockchain and the decentralized interplanetary le system (IPFS) on a mobile cloud platform | EHR | - Data breaches - Data tampering |
| Radhakrishnan et al. (2019) | This paper proposes a multilevel authentication-based scheme to protect the blockchain from the attacks. | EHR | - Data breaches - Data tampering - Other malicious attacks (e.g. impersonation, etc.) |
| Ramani et al. (2018) | This research proposes a blockchain based secure and efficient data accessibility mechanism for the patient and the doctor in a given health care system. | EHR/EMR | - Data breaches - Data tampering - Other malicious attacks (e.g. impersonation, etc.) - Lack of patients' control over their data - Lack of data integrity |
| Zhu et al. (2019) | The authors propose a cloud health resource-sharing model based on consensus-oriented blockchain technology and have developed a simulation study on breast tumor diagnosis | Classification model | - Misdiagnosis/overtreatment |
